# Patients' perceptions about diagnosis and treatment of chronic myeloid leukemia: a cross-sectional study among Brazilian patients: Percepções dos pacientes sobre diagnóstico e tratamento da leucemia mieloide crônica: estudo transversal entre pacientes brasileiros

**DOI:** 10.1590/1516-3180.2014.0001306

**Published:** 2014-11-07

**Authors:** Nelson Hamerschlak, Carmino de Souza, Ana Lúcia Cornacchioni, Ricardo Pasquini, Daniel Tabak, Nelson Spector, Merula Steagall

**Affiliations:** I MD, PhD. Head of Bone Marrow Transplantation Unit, Hospital Israelita Albert Einstein (HIAE) and Associação Brasileira de Linfoma e Leucemia (ABRALE), São Paulo, Brazil; II MD, PhD. Titular Professor of Internal Medicine, Universidade Estadual de Campinas (Unicamp), Campinas and Associação Brasileira de Linfoma e Leucemia (ABRALE), São Paulo, Brazil; III MD. Attending Physician, Instituto de Tratamento do Câncer Infantil (ITACI), Instituto da Criança (ICR), Hospital de Clínicas da Universidade de São Paulo (HC-USP) and Associação Brasileira de Linfoma e Leucemia (ABRALE), São Paulo, Brazil; IV MD, PhD. Senior Professor, Postgraduate Program on Internal Medicine, Universidade Federal do Paraná (UFP), Paraná and Associação Brasileira de Linfoma e Leucemia (ABRALE), São Paulo, Brazil; V MD, PhD. Director, Centro de Tratamento Oncológico (Centron), São Paulo and Associação Brasileira de Linfoma e Leucemia (ABRALE), São Paulo, Brazil; VI MD, PhD. Titular Professor, Universidade Federal do Rio de Janeiro (UFRJ), and Head of the Hematology Service, Hospital Universitário Clementino Fraga Filho (HUCCFF), Universidade Federal do Rio de Janeiro (UFRJ), Rio de Janeiro; and Associação Brasileira de Linfoma e Leucemia (ABRALE), São Paulo, Brazil; VII President, Associação Brasileira de Linfoma e Leucemia (ABRALE), São Paulo, Brazil

**Keywords:** Leukemia, myelogenous, chronic, BCR-ABL positive, Leukemia, myeloid, chronic-phase, Protein-tyrosine kinases, National health programs, Perception, Patient compliance

## Abstract

**CONTEXT AND OBJECTIVES:**

Chronic myeloid leukemia (CML) requires strict daily compliance with oral medication and regular blood and bone marrow control tests. The objective was to evaluate CML patients' perceptions about the disease, their access to information regarding the diagnosis, monitoring and treatment, adverse effects and associations of these variables with patients' demographics, region and healthcare access.

**DESIGN AND SETTING:**

Prospective cross-sectional study among CML patients registered with the Brazilian Lymphoma and Leukemia Association (ABRALE).

**METHODS:**

CML patients receiving treatment through the public healthcare system were interviewed by telephone.

**RESULTS:**

Among 1,102 patients interviewed, the symptoms most frequently leading them to seek medical care were weakness or fatigue. One third were diagnosed by means of routine tests. The time that elapsed between first symptoms and seeking medical care was 42.28 ± 154.21 days. Most patients had been tested at least once for Philadelphia chromosome, but 43.2% did not know the results. 64.8% had had polymerase chain reaction testing for the BCR/ABL gene every three months. 47% believed that CML could be controlled, but 33.1% believed that there was no treatment. About 24% reported occasionally stopping their medication. Imatinib was associated with nausea, cramps and muscle pain. Self-reported treatment adherence was significantly associated with normalized blood count, and positively associated with imatinib.

**CONCLUSIONS:**

There is a lack of information or understanding about disease monitoring tools among Brazilian CML patients; they are diagnosed quickly and have good access to treatment. Correct comprehension of CML control tools is impaired in Brazilian patients.

## Abstract (pt)

**CONTEXTO E OBJETIVOS:**

Leucemia mieloide crônica (CML) exige estrita adesão à medicação oral e ao monitoramento do sangue e da medula. O objetivo foi avaliar percepções de pacientes com leucemia mieloide crônica (LMC) sobre a doença, seu acesso à informação sobre diagnóstico, monitoramento e tratamento, efeitos adversos e a associação destes com dados demográficos, geográficos e de acesso a tratamento.

**DESENHO E LOCAL:**

Estudo prospectivo transversal realizado com pacientes de LMC cadastrados na Associação Brasileira de Leucemia e Linfoma (Abrale).

**MÉTODOS:**

Pacientes com LMC recebendo tratamento do sistema público de saúde foram entrevistados por telefone.

**RESULTADOS:**

Entre os 1.102 pacientes entrevistados, os sintomas mais frequentemente levando à busca de consulta foram fraqueza e fadiga. Um terço foi diagnosticado por exames de rotina. O tempo entre sintoma inicial e procura por ajuda foi de 42,28 ± 154,21 dias. A maioria foi testada pelo menos uma vez para o cromossomo Filadélfia, mas 43,2% não sabiam os resultados. 64,8% fizeram exame de reação em cadeia da polimerase para o gene BCR/ABL a cada três meses. 47% acreditavam que LMC pode ser controlada, mas 33,1% acham que não há tratamento. Cerca de 24% disseram que ocasionalmente interrompem o tratamento. Imatinibe associou-se com náusea, câimbra e dor muscular. Aderência auto-reportada associou-se significativamente com hemograma normal e positivamente com uso de imatinibe.

**CONCLUSÕES:**

Falta informação ou compreensão sobre monitoramento entre pacientes com LMC; eles recebem diagnóstico rapidamente e têm bom acesso ao tratamento. A correta compreensão das ferramentas de controle em LMC está prejudicada entre eles.

## INTRODUCTION

Treatment of chronic myeloid leukemia (CML) used to include bone marrow transplantation, hydroxyurea and therapeutic regimens based on interferon-alpha (IFN-alpha). About 10 years ago, imatinib mesylate, a derivative of phenyl-2-amino-pyrimidine that is a selective inhibitor of BCR-ABL tyrosine kinase, which induces hematological and cytogenetic remission in CML cases, became the treatment of choice.^1^ The average age of 50 years among patients originally affected by CML (median of 60), as well as the lack of histocompatible donors and the early and late risks, limits the option of bone marrow transplantation to a minority of patients.^2^–^4^ Imatinib mesylate is a drug with proven efficacy for treating patients with CML and is indicated as first-line medication for patients with Philadelphia chromosome (Ph+) positive CML (a chromosomal translocation associated with CML that is used in diagnosing the disease).^5^,^6^ Today, second-generation tyrosine kinase inhibitors such as dasatinib and nilotinib have also been shown to be efficacious as first-line therapy.^7^–^10^ Every Brazilian citizen has the right to receive imatinib mesylate for treatment of CML, on a cost-free basis, provided by the government. However, no study has investigated the access to these treatments in Brazil yet.

This chronic disease may cause significant changes to the daily lives of individuals and their families,^11^,^12^ since management of this disease involves strict daily compliance with oral medication and regular blood and bone marrow control examinations. 13,14 In the context of chronic diseases, cancer is seen in the popular imagination as a cause of rapid finitude and suffering, among other meanings. Although there are cultural differences in how the disease and patients' expectations regarding the physician's role are viewed,^15^ even without universal policies to inform and involve patients in decisions that affect their care,^16^ most patients want information about their diagnosis and want their families to also be informed, even when their illnesses are severe.^17^–^20^ This has proved to be an important therapeutic tool,^13^,^21^ and some authors have suggested that this information decreases the patient's sense of isolation and contributes towards mutual cooperation in the doctor-patient relationship.^21^

Hematology-oncology is a specialty that deals with patients who now can count on increased survival based on the proposed treatments – treatments that, of course, are not without risks or side effects, but are tolerable. However, few studies have been conducted in Brazil to determine patients' desire to participate in treatment decisions, and there have not been any studies specifically on CML patients that have assessed their access to treatment and the time required for a diagnosis to be reached. Since there may be significant differences in patients' perceptions of the quality and quantity of information received and in their participation in medical decisions among populations in different countries, it is important that studies like this should be conducted in Brazil.^17^

## OBJECTIVE

The purpose of this study was to evaluate Brazilian CML patients' perceptions about the disease, their access to information regarding the diagnosis, treatment and care received, adverse effects and relationships with doctors; and associations of these variables with patients' demographics, region and access to healthcare.

## METHODS

### Design, participants and location

This was a prospective, longitudinal study among patients with CML who were registered with the Brazilian Lymphoma and Leukemia Association (ABRALE). ABRALE is a civil society organization formed by patients with leukemia and lymphoma and their families, which provides information and support to patients, and educational programs, publications and events to healthcare professionals across the country. The study was approved by the Ethics Committee of the Albert Einstein Institute for Teaching and Research.

During the period between April 28, 2008, and February 8, 2010, all patients registered with ABRALE were surveyed by telephone about the care they had received for this disease. Patients were enrolled if they had a diagnosis of CML and were registered in ABRALE. Patients are registered with ABRALE through direct enrolment in the healthcare services where they are treated, in eight Brazilian state capitals (Rio de Janeiro, Curitiba, Goiânia, Recife, Porto Alegre, Belo Horizonte, São Paulo and Salvador) and in the city of Campinas (state of São Paulo). All the participants were interviewed 1 to 3 times, and were asked about their perceptions of the disease and its treatment, the drugs they were using and any adverse effects, and their relationships with their doctors. A questionnaire was specially designed for this study, including questions concerning the variables below.

Patients registered with ABRALE, i.e. patients undergoing treatment for CML who were receiving drugs through the Brazilian public healthcare system (SUS, Sistema Único de Saúde), were included. The telephone number in ABRALE'S records was used to contact patients as many times as needed until they had time to be interviewed. Deceased patients and those whose contact information in the database was out of date or incorrect were excluded. If patients could not or did not want to participate in an interview at the first contact (because they were busy or not feeling well), they were called at least one more time. Sometimes, they were called more than three times. If in the end they could not be reached, they were excluded from the study.

### Variables studied

The patients' demographic characteristics, such as age, education level, geographic location and access to healthcare services were checked. The symptoms that led patients to seek healthcare, their specialists, the tests that were requested and performed (i.e. how the disease was diagnosed), and the time that elapsed between symptoms and the diagnosis, were recorded. The treatments and procedures performed (medications taken and bone marrow transplantation performed) and adverse effects and access to healthcare services and medication were also recorded. In addition, the patients' knowledge and awareness of their disease and its prognosis and their relationships with their families and friends were analyzed.

### Interviews

Three nurses who had previously been trained for this task, with clarifications about CML and the treatments available, conducted the telephone interviews. In order to reach the interviewees at a time when the interview would be possible, they made at least three calls to the home or mobile phone numbers of the patients registered at ABRALE (i.e. if the interview could not take place at the first contact, a second and/or a third attempt was made).

The interviews were conducted by telephone using three questionnaires specially formulated for this study. During the first contact, which was made between April and July 2008, the patients' demographic variables and the first data about symptoms, diagnosis and treatment were assessed. During the second contact, between August 2008 and January 2009, the same patient reported whether his or her treatment had been modified and whether there had been any adverse effects or any difficulties. During the third contact, which occurred between March 2009 and July 2010, the patient was asked about his or her treatment, any tests performed and their psychological care. The interviews lasted about 15 to 20 minutes. The responses were entered into computerized databases.

### Statistical analysis

The characteristics of the patients and their disease were described using absolute and relative frequencies of the categories of interest. The chi-square or likelihood ratio test was applied to determine whether there was any association between the categorized measurements of interest. The Kruskal-Wallis test was performed to compare demographics with questions related to treatment adherence. For statistical analysis, the Statistical Package for the Social Sciences (SPSS), version 15.0, was used.

## RESULTS

### Patients and care

During the study period, 1,102 patients with CML were interviewed. The patients' average age at the time of the interview was 47.8 years. The average age at diagnosis was 42 years (range: 3 to 84 years).

Most patients (75.86%) were from the southern and southeastern regions of Brazil. Although all of them were entitled to free care through SUS in Brazil, and were even receiving the cancer treatment drugs through the Brazilian public healthcare system, 44.8% of the respondents also had a private health plan or insurance. Most of these were in the southeastern region (P < 0.001), whereas the remainder depended solely on public assistance.

Just over half (51.4%) of the respondents had had to travel to another city to get specialized care. Most of the Brazilians who had had to travel to receive treatment were living in the northern region (73.3% had to travel) and southern region (63.3% had to travel). The region of residence was significantly associated with the need to travel (P < 0.001).

### Diagnosis and follow-up

The first symptoms that most frequently led the respondents to seek medical help were weakness or fatigue (45.5%), weight loss (36.3%) and bone pain (22.4%). Only 9.1% had fever. One third (33.8%) of the patients discovered that they were sick from tests that were usually performed due to other clinical suspicions, and 4.5% had anemia.

The time that elapsed from experiencing the first symptoms to seeking initial medical help varied widely, with an average of 42.28 ± 154.21 days. There was no association between this interval and the patient's region of residence. The time that elapsed between the first visit to a general practitioner and a consultation with a specialist was also checked. The median time was three days, but the variation was wide. Again, there was no association between the elapsed time and the geographical region.

The patients were asked whether they had been tested for the presence of the Philadelphia chromosome after beginning treatment for CML, and the responses are shown in Table 1. Most of the patients had been tested at least once. Twenty percent of the patients did not answer the question regarding the frequency with which they were tested. Among those who responded, most stated that they had been tested for the Philadelphia chromosome once (33.3%) or twice (34%) yearly. A small proportion (5.8%) said they had been tested 4 times yearly. The remaining patients stated that they had been tested once every two years (5.4%), did not know the frequency of testing (13.3%), or that "they were no longer tested" (8.2%), meaning that they had probably only been tested once.

**Table 1:** Patients' responses to questions about the date of the Philadelphia chromosome test

93.3% had been tested	33.6% did not recall the date
	21.8% up to six months ago
	35.1% six months to one year ago
	2.8% over one year ago
5.1% had not been tested	2.3% indicated the reason as "recent diagnosis"
	2.8% indicated no reason at all
1.6% had never heard of the test	

Among the interviewees, 43.2% could not say whether the Philadelphia chromosome test became negative after they had taken the medication, 36.1% reported that the test became negative and 20.8% that it was non-negative. There was no association between knowledge of the test result and the participant's geographical region.

The patients were also asked whether they had had the polymerase chain reaction (PCR) test to count the number of copies of the BCR/ABL gene; 64.8% of respondents revealed that they had had the test every three or six months, or every year. A few said they had never had the test (1.2%) or that they had had the test "every month" (6.6%), and 26.3% stated that they did not know about the PCR test. Again, there was no significant association between knowledge or frequency of the test and the participant's geographical region.

In order to investigate the frequency of monitoring with a complete blood count (CBC), the participants were asked when they had last had this test. The vast majority (92.9%) had undergone blood counts one, two or three months previously. Others (7.1%) reported that more than three months had elapsed since having the test. The vast majority reported that "the blood test was normal" as a result of taking the drug (80.7%), but 13.8% said that their test was not normal and 5.4% said that they did not know the test result.

### Perceptions of the disease and its treatment

Most participants knew something about the disease before receiving their diagnosis of leukemia (67.5%) or had cases in the family (6.2%). However, 26.3% had never heard of the disease.

The survey also addressed the relationship between patients and their doctors. Most patients said that they "always" (76.1%) or "sometimes" (15.6%) asked the doctor questions to clarify their doubts. Only a small portion (2.5%) stated that the consultation time was too short to ask questions or that they had difficulty understanding the doctor (2%). Those who answered that they did not have a good relationship with their doctor sought other means of informing themselves (3.8%).

Most of the patients relied on the treatment. Most believed that "there is a treatment for CML and, if followed, the disease will be controlled" (47%), or even that after some time on treatment "the person is cured" (19.9%). But 33.1% were skeptical; they believed that there was no treatment to even control the disease. There was no association between these responses and the frequency with which the drugs were taken.

The diagnosis of leukemia improved half (50.7%) of the patients' relationships with family and friends, whereas it remained the same for 42%. Only 7.3% said that the relationship with other people had worsened. There was an association between little emotional support received by patients from their family and a feeling among patients that that the diagnosis had worsened their relationship with their family and friends (P = 0.001). Situations in which patients were close to their partners were also associated with receiving support from their family (r = 0.522, P = 0.0001) and with being satisfied with their sex life (r = 0.544, P = 0.0001).

Being concerned about possible worsening was a response correlated with the fear of having new disease symptoms (r = 0.582, P = 0.0001). On the other hand, patients who said they were able to work felt fulfilled with the work they performed (r = 0.611, P = 0.0001) and were also able to perform their usual activities (r = 0.601, P = 0.0001).

Diet did not change for 61.5% (although 52.7% reported experiencing changes in appetite); 71.2% did not cease to do physical activities, and 57.7% did not interrupt their work.

Undergoing bone marrow transplantation was positively associated with the response to the question of how the patients faced the disease (P = 0.002) and how much pleasure in life they felt (P = 0.004), thus revealing an association between these three aspects of the patients. Transplantation also was significantly associated with a change in eating habits (P < 0.01) and having a good appetite (P = 0.030).

Most patients (74.4%) believed that their health had improved with the various treatments that had been applied; 6.9% thought it had worsened, and 18.7% saw no difference. A portion of them (23.3%) reported that their health had worsened at some point during their therapy.

Among the 1,097 respondents, 78.3% believed that their attitude could influence the result of their treatment, and 3.2% thought that only some attitudes interfered. On the other hand, 18.3% of the patients believed that no action of theirs could interfere with their treatment. The response to the question regarding the interference of certain actions with success of treatment was not significantly associated with reports of disruption in taking medication. During the first interview, only 6.5% of the patients admitted that they had discontinued their medication at some time on their own. However, 24.4% revealed that they had stopped taking their medication on some days (failure to take the medication or running out of the drug), and 24.0% stated that this had occurred during the past month.

As already stated, every Brazilian citizen has the right to receive medication for treatment of CML, on a cost-free basis, and this is provided by the government. As shown in Table 2, most patients were on imatinib during the three stages of the study. The use of the different treatments did not change much over time; 52.2% of the patients said that they were currently taking drugs that had been prescribed for them for two or more years, and 15% for at least one year.

**Table 2:** Average proportions of treatments reported as used during the three interviews (n = 1102)

Treatment	Research n (%)
Imatinib	801.67 (72.73%)
Bone marrow transplantation	82.67 (7.50%)
Tyrosine kinase inhibitors, second generation	84.67 (7.67%)
Interferon	14.67 (1.33%)
None	26.0 (2.35%)
Others	94.50 (8.50%)

Most patients (75.95%) said that they had not experienced difficulties in receiving their medication from the government for treatment of CML. The least difficulty was reported in the southern and southeastern regions (P < 0.01; likelihood ratio test). Most (79.2%) also had not had any difficulty receiving the drugs during the month preceding the survey (again, with a predominance of easy access in the southern and southeast regions; P < 0.001).

Among 243 patients who said that they had failed to take the drug at some time in the past month, 90 (8.2% of the total sample) said that this was due to forgetfulness, 38 (3.4%) due to medical advice, and 81 (7.4%) because of a lack of the medicine, with no information available about the reason for the remaining 34 patients. On average, these patients went 10 days without taking the drug (median: 3 days); 22% said that they had not paid proper attention to the schedule for taking the medicine, and 3% admitted to having reduced or increased the doses on their own.

In most cases, imatinib was associated with the following side effects: nausea (54.6%, P < 0.001), cramps (65.4%, P < 0.001) and muscle pain (52.3%, P < 0.001).

As shown in Table 3, there was a significant association between continuity in taking the prescribed medication (self-reported) and normalization of blood counts. This correlation was positive with the drug imatinib (i.e. between use of the drug and normalization of the test).

**Table 3:** Association between normal results in the complete blood count (CBC) and treatments in patients with chronic myeloid leukemia

		Was your CBC (hemogram) test normal?								
		Yes		No		Don't know		Total		P
		n	%	n	%	n	%	n	%	
At the last interview you said you were taking ........]. Are you still taking it?	Yes	809	91.1	126	82.9	48	80.0	983	89.4	**0.001**
	No	79	8.9	26	17.1	12	20.0	117	10.6	
	Total	888	100.0	152	100.0	60	100.0	1100	100.0	
Bone marrow transplantation	No	821	92.4	144	94.7	52	86.7	1017	92.4	0.161*
	Yes	68	7.6	8	5.3	8	13.3	84	7.6	
	Total	889	100.0	152	100.0	60	100.0	1101	100.0	
Aracytin (chemotherapy)	No	888	99.9	151	99.3	60	100.0	1099	99.8	0.445*
	Yes	1	0.1	1	0.7	0		2	0.2	
	Total	889	100.0	152	100.0	60	100.0	1101	100.0	
Dasatinib	No	831	93.5	136	89.5	56	93.3	1023	92.9	0.240*
	Yes	58	6.5	16	10.5	4	6.7	78	7.1	
	Total	889	100.0	152	100.0	60	100.0	1101	100.0	
Interferon	No	883	99.3	146	96.1	60	100.0	1089	98.9	**0.007** *
	Yes	6	0.7	6	3.9	0		12	1.1	
	Total	889	100.0	152	100.0	60	100.0	1101	100.0	
Imatinib	No	225	25.3	63	41.4	27	45.0	315	28.6	**< 0.001**
	Yes	664	74.7	89	58.6	33	55.0	786	71.4	
	Total	889	100.0	152	100.0	60	100.0	1101	100.0	
Hydroxyurea	No	866	97.4	132	86.8	54	90.0	1052	95.5	**< 0.001** *
	Yes	23	2.6	20	13.2	6	10.0	49	4.5	
	Total	889	100.0	152	100.0	60	100.0	1101	100.0	
Nilotinib	No	850	95.6	143	94.1	56	93.3	1049	95.3	0.569*
	Yes	39	4.4	9	5.9	4	6.7	52	4.7	
	Total	889	100.0	152	100.0	60	100.0	1101	100.0	
None	No	866	97.4	147	96.7	56	93.3	1069	97.1	0.269*
	Yes	23	2.6	5	3.3	4	6.7	32	2.9	
	Total	889	100.0	152	100.0	60	100.0	1101	100.0	
Other	No	874	98.3	150	98.7	58	96.7	1082	98.3	0.640*
	Yes	15	1.7	2	1.3	2	3.3	19	1.7	
	Total	889	100	152	100	60	100	1101	100	

Results from the association tests, chi-square test or

* likelihood ratio test.

## DISCUSSION

This was the largest study on patients' perceptions of CML diagnosis and treatment in Brazil, interviewing 1,102 patients.

The goal in this study was to evaluate how treatment is distributed in the country and the difficulties faced by patients with regard to access to treatment and information about the disease. We found that the majority of the patients lived in the southeastern region of Brazil, where the treatment of CML is still heavily concentrated. Although public healthcare in Brazil is distributed throughout the country, treatments of high complexity still tend to be concentrated in the southeastern region. Particularly for CML, the expanded access program for imatinib began in centers located in this region, and many patients maintained their treatment there, despite widespread free distribution of the drug by the federal government in all states.^22^

This study found that fatigue or weakness was the symptom most frequently leading patients with CML to seek medical help and have the disease diagnosed. In another study showing the characteristics of CML patients during their first visit to healthcare services, 40% were asymptomatic; however, among the symptomatic patients, fatigue, anorexia and weight loss were typical symptoms. For 40% of those asymptomatic patients, the diagnosis was made from abnormal granulocytic counts seen in the blood analysis, which were present in up to half of the patients.^23^ Perhaps the use of a symptom as nonspecific as fatigue explains why there are huge variations in the time that elapses from experiencing the first symptoms to seeking medical help, as seen in the present study. While some patients sought help within the first days after the appearance of symptoms, others took months to do so, and it was not possible to establish a pattern of behavior in this study. The onset of symptoms in a chronic disease like CML occurs at varying times, which may explain this finding.

A major problem today in using tyrosine-kinase inhibitors is adherence: these drugs are able to adequately control the disease, but with the need for medicine to be taken daily. A recent study on the impact of adherence to imatinib on survival found that, at some point during treatment, 29.6% of the patients were considered to be nonadherent (using the criterion of discontinuation of treatment, without any medical prescription for more than one week). These nonadherent patients were more likely to fail to achieve a complete cytogenetic response and had shorter survival.^24^ Among patients with CML treated with imatinib for some years, poor adherence may be the predominant reason for inability to obtain adequate molecular responses,^25^–^27^ i.e. a reduction in BCR-ABL1 transcripts, or a cytogenetic response.^25^,^26^ Treatment adherence to imatinib should be monitored routinely^28^,^29^ because noncompliance leads to poor response to treatment and higher costs.^25^,^28^,^30^–^32^ Treatment interruptions (i.e. failure to refill the imatinib prescription) can be detected by many adherence measurement tools in current use for imatinib: the medication possession ratio (MPR),^27^,^28^,^32^ retrieval of medication from the healthcare services providing them,^25^,^33^ or simply by monitoring plasma levels.^31^,^34^ Therefore, blood samples should be taken regularly in order to evaluate disease control and medicine intake.

One important finding of this study was the poor understanding that the patients had about the diagnostic and monitoring tools for the disease. Information is only one of the many challenges facing leukemia patients and their caregivers, as recently shown.^12^ For example, although most patients in our study remembered being tested for the Philadelphia chromosome, only 20% knew the frequency of testing, 33.6% did not know the date of the last test and many did not know what the result of the test was. This finding is interesting because the test for the Philadelphia chromosome involves collecting bone marrow (for a myelogram), which usually does not go unnoticed. Although bone puncture is always done under anesthesia, it is an unusual and uncomfortable examination that is difficult to forget. Karyotyping is necessary in order to monitor the status of the Philadelphia chromosome and, in patients with CML, should be performed periodically.

In a study on physicians affiliated to the Latin America Leukemia Net (LALNET), 72% stated that they did bone marrow karyotyping to monitor their patients, 54% said that they performed the test every six months, and 31% said that they performed the test every four months.^35^ In Brazil, as seen in this study, patients reported a curious lack of information or difficulty in understanding the schedule for the monitoring of their disease. We found that the same "misinformation" occurred with the PCR test; 26.3% of the patients in this study were not familiar with the test. The LALNET study showed that 41% of Latin American doctors prescribed the PCR test every six months and 31% every three months.^35^ Considering that the physicians reported complying with the international monitoring recommendations, the finding in this study may reflect a lack of understanding among patients regarding the monitoring schedule (for example, confusion between CRP and CBC, which are both evaluated by collecting venous blood) and regarding the usefulness of each test, despite the efforts of the healthcare providers and associations such as ABRALE to educate this population about their illness and its treatment. A multidisciplinary approach may be a good tool for dealing with this challenge.^13^

While demonstrating less knowledge than they should about their own health monitoring, the patients interviewed seemed satisfied with the doctor-patient relationship. Only 2% said that the consultation time was too short. These responses are contradictory, since they indicate an impossible scenario; patients say they are satisfied with their relationship with their doctor and that the consultation time is satisfactory, but they do not know when the last karyotyping for detection of the Philadelphia chromosome occurred.

One possible explanation could be that the language used by the doctor might not be effective in communicating with the patient. Another could be that the patients themselves have little interest in finding out about their own disease. The emotional aspects of CML patients were also addressed by the survey, and 33.1% were skeptical and believed that there was no treatment to even control their disease. Possibly, some of these patients do not seek information from their doctor or other sources, owing to their disinterest and lack of hope. Nonetheless, these speculations must be investigated more appropriately.

Another study on adherence to chemotherapy mapped the steps involved in complex processes like adherence, including the prescription, drug delivery, administration and phases of drug use, also including reports of adverse effects made by patients. "Make lists to guide and remind clinicians about the key elements of patient education" was the authors' main recommendation with regard to imatinib, and to "offer patients and their families educational materials about the protocols, and a phone number to resolve questions".^36^ In fact, there is already a consensus that proper education of patients can reduce the risk of noncompliance,^28^,^30^,^32^ and consequently reduce the economic burden of CML. Additionally, the patient is the main guardian of his/her treatment and disease monitoring: the results from treating CML depend not only on taking the medicine appropriately but also on clinical decisions based on hematological, cytogenetic and molecular controls.

The factors known to predict adherence to therapy include information about the disease,^37^–^39^ frequent contact with a single hematologist, easy access to the treatment clinic^38^,^39^ and participation in decision-making about the disease and treatment.^38^ A study has shown that patients felt inappropriately reassured by physicians that their nonadherence would not have a detrimental effect on their clinical response.^40^ Therefore, information about the patients' perceptions and their level of knowledge on the subject is very useful for clinicians and will certainly help in managing the disease, quality of life and treatment outcomes.

Our study was conducted in three stages. Although we did not evaluate adherence directly, we had the opportunity to ask patients about their use of medication. The proportions of patients using different CML treatments had not changed significantly over time, with most using imatinib. Few patients in this study reported forgetfulness or admitted that they intentionally stopped taking the medicine.

In Brazil, CML is diagnosed quickly. Patients have good access to treatment and understand that although the disease is not curable, it can be controlled with medication. Many believed that the occurrence of the disease helped improve their relationships with family and friends. However, there are still small proportions of patients who remain skeptical about the effectiveness of the treatment.

From the results of this study, we recommend that patients be given information about the importance and significance of periodic tests to monitor CML, since many are still ignorant of essential tests like that for the Philadelphia chromosome.
